# Synchronous Ipsilateral Pleomorphic Adenomas of Parotid and Submandibular Glands: An Unusual Finding

**DOI:** 10.1155/2020/8887867

**Published:** 2020-12-28

**Authors:** Walter Colangeli, Aleksandr Kapitonov, Valerio Facchini, Marta Zappalà, Evaristo Belli

**Affiliations:** U.O. Maxillofacial Surgery, S. Andrea Hospital, La Sapienza University, Rome 00189, Italy

## Abstract

A rare case of synchronous ipsilateral pleomorphic adenomas (PA) of the left parotid and submandibular glands is reported. Simultaneous multiple PA in major salivary glands are a very rare entity, and merely few cases of ipsilateral synchronous PA involving parotid and submandibular glands are reported in the literature. The case of a 40-year-old female with a six-year history of asymptomatic growing lesion in both left parotid and left submandibular regions is presented. Left superficial parotidectomy and left submandibular gland excision at the same surgery have been performed. The aim of this article is to highlight the importance of an accurate head and neck presurgery examination both clinically and radiologically, keeping in mind the possibility of multiple tumor location.

## 1. Introduction

Among head and neck neoplasms, salivary gland tumors are around 2.0–6.5% [1].

Pleomorphic adenoma, a benign mixed tumor with both epithelial and connective components, is the most common benign neoplasm occurring in the salivary glands [2–4].

Mostly located in the parotid gland (about 80%), it may originate from the submandibular gland and seldom from sublingual and minor salivary glands [5, 6].

A single nodular expansive lesion located in one of these glands is the typical presentation, while in 3.4% of the cases, multiple masses are found. While Warthin's tumor is the most common histotype found in multiple salivary gland neoplasm, only few cases of multiple pleomorphic adenoma (MPA) are reported in the literature [7–13].

Bilateral parotid location is the most common presentation of MPA. Synchronous unilateral pleomorphic adenomas involving both parotid and submandibular glands are extremely rare, and merely ten cases have been reported in the literature till date [14–25].

Herein, a case of synchronous multiple pleomorphic adenoma affecting both the parotid and ipsilateral submandibular glands is presented.

Considering this uncommon possibility, our findings confirm the importance of meticulous presurgery examination when a single salivary gland tumor is detected.

## 2. Case Report

In March 2019, a 40-year-old female was admitted to our Maxillo-Facial Surgery Department, at “La Sapienza” University, Sant'Andrea Hospital of Rome, Italy, with two voluminous masses in the left preauricular and left submandibular regions. Her past medical history was well assessed.

She first noticed these lesions about 6 years ago, when they were the size of a bean. The gradual growth was referred by the patient and documented by annual ultrasonography exams. Three years ago, she performed an FNAC that was suggestive for pleomorphic adenoma. Finally, she presented to our attention.

No history of pain, fever, trauma, difficulty in mouth opening, or facial nerve weakness was referred. No risk factor like smoking or previous radiation exposition was found.

Physical examination revealed two solid masses, one in the left preauricular parotid region and the second one in the left submandibular region, both well-circumscribed, hard-elastic, not fixed to the adjacent anatomic structures. No enlarged cervical lymph nodes were detected. There was no evidence of facial and trigeminal nerve dysfunction, and the skin overlying masses was normal.

**Figure 1:**
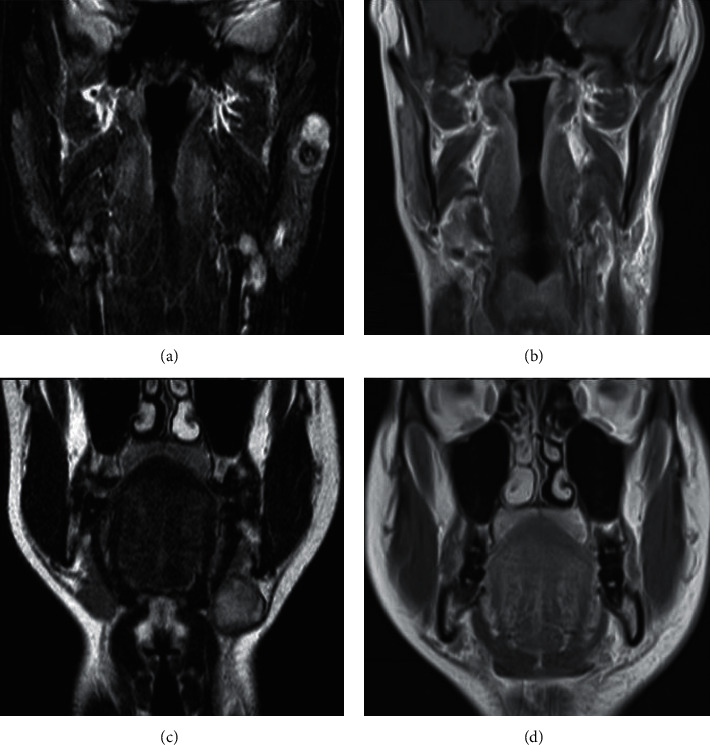
(a, c) Presurgery contrast-enhanced MRI of the head and neck; (b, d) 3-month follow-up MRI.

A contrast-enhanced MRI of the head and neck was performed and showed a well-defined slightly lobulated mass (20 × 25 mm) with inhomogeneous peripheral enhancement in the left parotid gland and a second mass with well-defined nodular aspect (30 × 26 mm) and central inhomogeneous enhancement in the left submandibular gland (Figure 1).

In May 2019, right-sided superficial parotidectomy and right submandibular gland excision at the same surgery were performed.

A preauricular and pretragal incision with cervical extension was executed to obtain a complete exposure of both parotid and submandibular glands.

Moderate weakness of mandibular branches of the left facial nerve was early evaluated after surgery. Complications were not observed, and the postsurgical period was regular.

**Figure 2:**
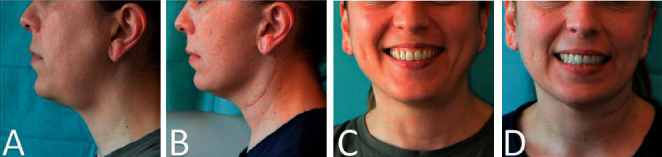
(a, c) Clinical view of ipsilateral swellings of left parotid and submandibular regions; (b, d) 3-month follow-up clinical view.

A 3-month follow-up revealed complete recovery of facial nerve weakness (Figure 2).

**Figure 3:**
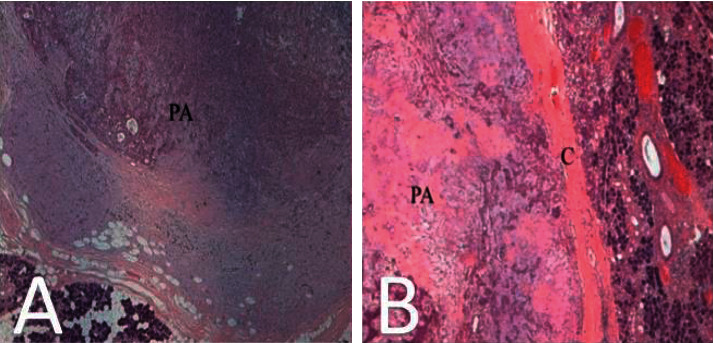
Histological view of pleomorphic adenoma of the parotid gland (a) and of the submandibular gland (b). PA = pleomorphic adenoma; C = capsule.

Histopathological evaluation confirmed the benign pleomorphic adenoma diagnosis of both lesions (Figure 3).

## 3. Discussion

Multiple salivary gland tumors are a rare entity observed only in 3.4% of cases. Warthin's tumor is the most common histotype while uncommon cases of multiple pleomorphic adenoma (MPA) are reported in the literature [7–13].

MPA usually affect both parotid glands, and a unilateral localization involving both parotid and submandibular glands is a very unusual event; merely ten cases have been reported in the literature till date [14–24].

A large amount of theories were discussed about the origin of these multiple tumors both considering these lesions as two unrelated primary sites of tumor and multiple foci of a low-grade malignant neoplasm [23, 26–28].

According to the last theory, multiple pleomorphic adenomas are considered as lesions with low-grade malignant potential with poorly understood pathogenesis, also known as metastatic pleomorphic adenoma [23].

In 1953, Foote and Frazell [29] first described metastatic pleomorphic adenoma, and in 2005, the WHO (World Health Organization) defined it as a “histologically benign pleomorphic adenoma that inexplicably manifests local or distant metastasis” [30]. However, in 2017, this definition was delated from the classification.

According to Alshgroud et al. when considering MPA, it is important to evaluate the age of the patient, the location of the presumed metastatic lesion, the number of recurrences, the period between the primary and metastatic lesions, the possibility of iatrogenic spread, the histopathology of both lesions, the possibility of the presence of other metastatic lesions, and any chromosomal or molecular similarities between the two lesions [23].

Considering that metastatic lesion is most located nearby the previously operated surgical site, Singh et al. hypothesized that this new lesion could be a result of tumor spreading through the lymphovascular system at the time of primary excision, even if the possibility of metastasis from one gland to the other cannot be excluded yet [24].

Klijanienko et al. suggested that metastasizing pleomorphic adenomas may represent unrecognized malignancy with 20% death rate associated [31].

Regarding another risk factor for multiple pleomorphic adenomas, radiation was described by Nagler and Laufer [17].

Although our patient was not exposed to radiations nor presented history of surgery or trauma or any other risk factor, she has developed multiple pleomorphic adenoma.

For all salivary gland neoplasms, single or multiple, unilateral or bilateral, the treatment of choice is complete excision of the lesion with adequate resection margins. In the case of parotid tumors, a superficial parotidectomy or total parotidectomy depending on the location and sizes of the tumor should be performed. However, in submandibular gland tumors, the removal of the whole gland is suggested to ensure complete disease removal [32, 33].

Superficial parotidectomy is no risk free, and transient or permanent facial nerve paralysis, Frey's syndrome, salivary fistula or subcutaneous effusion, infection, xerostomia, scar deformity, great auricular analgesia, hematoma, and seroma may occur [34, 35].

Furthermore, submandibular gland excision's complications include transient or permanent palsy of the mandibular branch of the facial nerve, transient or permanent palsy of the lingual nerve, hematoma, wound infection, and salivary fistula [33].

Bearing in mind that the occurrence of these complications is mostly related to the postoperative period and to the surgery procedure, performing simultaneously submandibular excision and superficial parotidectomy reduces, but not eliminates, the net risk of surgical site or wound infection and hematoma.

The preauricular and pretragal incision with cervical extension permits to obtain a complete exposure of both parotid and submandibular glands giving the surgeon great operative field visibility with a good aesthetic outcome.

Moreover, in case of a single surgery, the intrinsic risks of general anaesthesia are reduced compared to the risk of two separate surgeries.

On the other hand, single surgery is more invasive and requires adequate surgical experience and a careful postoperative follow-up.

Despite that, considering the numerous advantages of the technique, the authors strongly recommended simultaneous ipsilateral parotidectomy and submandibular gland surgery for synchronous ipsilateral parotid and submandibular gland tumors.

## 4. Conclusions

Our case and the literature review highlighted the importance for an accurate head and neck presurgery examination, including clinical patient history, risk factors, tumor location, its histological typing, and the evaluation of other salivary glands with radiological exams keeping in mind the possibility of multiple tumor location.

In these cases, postsurgery follow-up is becoming of particular importance for early possible multiple foci and recurrence detection.
